# African Swine Fever: Transmission, Spread, and Control through Biosecurity and Disinfection, Including Polish Trends

**DOI:** 10.3390/v15112275

**Published:** 2023-11-19

**Authors:** Małgorzata Juszkiewicz, Marek Walczak, Grzegorz Woźniakowski, Katarzyna Podgórska

**Affiliations:** 1 Department of Swine Diseases, National Veterinary Research Institute, Partyzantów 57 Avenue, 24-100 Puławy, Poland; marek.walczak@piwet.pulawy.pl (M.W.); katarzyna.podgorska@piwet.pulawy.pl (K.P.); 2 Department of Diagnostics and Clinical Sciences, Faculty of Biological and Veterinary Sciences, Nicolaus Copernicus University in Toruń, Lwowska 1 Street, 87-100 Toruń, Poland; grzegorz.wozniakowski@umk.pl

**Keywords:** African swine fever, disinfection, biosecurity

## Abstract

African swine fever is a contagious disease, affecting pigs and wild boars, which poses a major threat to the pig industry worldwide and, therefore, to the agricultural economies of many countries. Despite intensive studies, an effective vaccine against the disease has not yet been developed. Since 2007, ASFV has been circulating in Eastern and Central Europe, covering an increasingly large area. As of 2018, the disease is additionally spreading at an unprecedented scale in Southeast Asia, nearly ruining China’s pig-producing sector and generating economic losses of approximately USD 111.2 billion in 2019. ASFV’s high resistance to environmental conditions, together with the lack of an approved vaccine, plays a key role in the spread of the disease. Therefore, the biosecurity and disinfection of pig farms are the only effective tools through which to prevent ASFV from entering the farms. The selection of a disinfectant, with research-proven efficacy and proper use, taking into account environmental conditions, exposure time, pH range, and temperature, plays a crucial role in the disinfection process. Despite the significant importance of ASF epizootics, little information is available on the effectiveness of different disinfectants against ASFV. In this review, we have compiled the current knowledge on the transmission, spread, and control of ASF using the principles of biosecurity, with particular attention to disinfection, including a perspective based on Polish experience with ASF control.

## 1. Introduction

Pig production in Poland is one of the most important branches of livestock farming and, therefore, contributes significantly to the country’s food economy. In 2017, pork production accounted for more than 35% of total meat production, second only to poultry production (over 48%). According to data from the Central Statistics Office, the pig population in December 2021 amounted to 10,242.4 thousand heads, showing a decrease of 12.7% compared to the same period in the previous year. This population reduction occurred in all pig production groups, particularly in the sow herd group, where it amounted to 20.6% [1]. One of the main reasons for the lack of profitability in pig production in Poland is the occurrence of infectious diseases, especially those controlled ex officio, including African swine fever (ASF). 

ASF is one of the most dangerous contagious viral diseases affecting pigs and wild boars. Although ASF spreads slowly, unlike classical swine fever (CSF), the disease consistently expands its reach. In a herd of infected pigs, the disease affects a significant percentage of individuals, but not the majority of animals, which makes diagnostic investigations complicated at the early stages of ASF [2]. In a population of infected wild boars, ASF spreads at a rate of 10–12 km per year, in the absence of an additional “human” factor, which, among other factors, plays a key role in the advancement of the disease over long distances (up to several hundred kilometres) in Poland and other epizootic countries [3].

ASF is subjected to official control by and notification to the World Organisation for Animal Health (WOAH) [4]. The disease affects the global economy for many pig- and/or pork-exporting countries, as well as local pig production. For example, China, which is a world leader in pork production (54% of pork carcasses in 2017) [5,6], experienced an overall 41% decrease in its pig population between 2018 and 2019, as a result of ASF occurrence [7].

Eight years of ASF epizootics in Poland were initiated in 2014, with wild boar outbreaks close to the border with the Republic of Belarus, and the subsequent spread of the disease within the wild boar population, as well as outbreaks on pig farms. As a result of the struggle to control the disease, the approach to pig farming within the country has changed significantly. This change involves the liquidation of many small-scale and backyard pig farms, where it was not possible to introduce the required biosecurity measures [1,8]. It is worth mentioning that, in 2015, there were about 250,000 pig farms in Poland; in 2021, only about 84.7 thousand remained. Despite the passage of time, and the increased awareness among breeders regarding the principles of biosecurity as the main element to protecting pig herds against ASF, there has been no clear improvement in the epizootic situation within the country. According to data compiled by analysts from Gobarto S.A., in the first quarter of 2021, approximately 23,000 jobs were liquidated in Poland, accounting for more than 20% of national breeding; meanwhile, in the first four months of 2021, the pig population decreased by 10% compared to the population in January (2021) and amounted to approximately 10.2 million heads [9]. According to an analyst in the agri-food sector, ASF-related restrictions on pork exports from Poland, and the consequent loss of important export markets (e.g., China, South Korea, and Japan), resulted in a 14% reduction in foreign sales of pork. This reduction has led to an approximate average loss of nearly EUR 62.7 million per year within this sector of the economy [9]. 

The complex structure, genotypic diversity, and ability of ASFV to adapt to the environment pose significant problems for vaccine development, making it difficult to control the spread of the disease. Due to the lack of an effective and safe vaccine, as well as a lack of effective treatment methods, ASF is continually decimating pig herds in affected countries, causing huge economic losses. Considering the importance of pork in domestic meat production, maintaining an appropriate level of pig herd production is a strategic objective of the food economy, which can be ensured by maintaining a sufficiently high level of welfare and biosecurity on pig farms. Currently, it seems necessary to educate pig farmers about the requirements for biosecurity and proper disinfection.

## 2. Historical Outline and Current Epizootic Situation

A highly contagious and deadly disease of Kenyan pigs, designated as ASF, was first described by Eustace Montgomery in 1921 [10]. For years, ASF was found only in sub-Saharan Africa, but in 1957, through human activity, it was introduced to Portugal. This was the first introduction of the disease to Europe, for which genotype I of the ASF virus (ASFV) was responsible [11]. In the following years, ASF was found in other countries: Spain (1960), France (1986), Italy (1967, 1969, and 1993), Malta (1978), Belgium (1985), and the Netherlands (1986). It took almost 40 years to eradicate the disease from the Iberian Peninsula [4,12]. Since the first introduction of the disease to Europe, ASF has been endemic in Sardinia. In 2021, Sardinia’s president, Francesco Pigliaru, declared the island ASF free [13], considering the success of eradicating the disease by controlling the free-range Brado pig farms, which were the reservoirs of ASFV (genotype I). Unfortunately, in January 2022, in northwest Italy, ASFV (genotype II) was confirmed in a dead wild boar in the Piedmont region. This outbreak was approximately 800 km from the nearest outbreaks of this disease in Europe (eastern Germany) and was followed by three new outbreaks [14]. Unfortunately, recent reports have confirmed the first outbreak of ASF (genotype II) among pigs on the Dorgali farm in Sardinia [15].

After the first epizootics in Europe, the next wave of ASF (genotype I) outbreaks started in 2007 in Georgia. The probable cause of the introduction of ASFV from East Africa was the transport of pigs or waste food contaminated with the virus, which was fed to the pigs after it reached the port of Poti in Georgia [16]. From there, the virus quickly spread to other countries, including Armenia, Azerbaijan (2007), Russia (2008), Ukraine (2012), and Belarus (2013) [17,18,19]. Then, in 2014, its presence was confirmed within the European Union, including in Lithuania, Latvia, Estonia, and Poland. In the following years, the disease spread to Romania (2017), the Czech Republic (2017), Moldova, Belgium, Bulgaria (2018), Slovakia (2019), Serbia (2019), Germany (2020), Greece (2020), and more recently, to the Dominican Republic and Haiti (2021); thus, after more than 40 years of absence, the virus reached the Americas. In 2022, ASF was also confirmed in continental Italy, after 40 years of absence in this area. The first occurrences of the disease in North Macedonia and Thailand were reported in January 2022, and in Nepal in March 2022. Currently, ASF has been confirmed for the first time on domestic pig farms in Bosnia and Herzegovina, Croatia, and the Republic of Kosovo, as well as in wild boars and imported live pigs in Singapore [11,17,20,21,22].

In 2018, the first outbreaks of the disease were reported in Asia, specifically in Chinese territory. This initiated an epizootic spread of the disease to other neighbouring countries: Cambodia, Hong Kong, Indonesia, Vietnam, Laos, Mongolia, Taiwan, North Korea, South Korea, Myanmar, East Timor, the Philippines, and areas of Russia bordering the People’s Republic of China (PRC). The consequences of an ASF outbreak in the PRC have been catastrophic, both for the domestic economy and for international trade. The estimated contribution of the PRC to global pork production in 2018 was 45% of the total number of pigs in the world. However, after the outbreak of the disease, there was a 40% decrease in the pig population, marking the first decline in this sector of the economy in 20 years [7,23]. Within a year of ASF detection in the PRC, there were 162 ASF outbreaks in pigs, during which 13,355 pigs died from the disease and 1,204,281 animals were killed during outbreak eradication. The total economic loss in the PRC due to the epizootic ASF was estimated at approximately USD 111.2 billion in 2019 alone [24]. 

Since 2005, the disease has been recorded in more than 74 countries in Africa, Europe, and Asia, and has also reappeared on the American and Oceanic continents [22,25]. According to a WOAH report published in 2021, a total of 36 countries remain in the epizootic state of ASF, including 13 in Europe, 16 in Asia, 3 in Africa, 2 in Latin America, and 2 in Oceania [25,26]. To date, only two countries in the European Union (EU) have successfully eradicated ASF in their wild boar populations. The last ASF outbreak in wild boars in the Czech Republic was detected in April 2018, while in Belgium, the last outbreak was found in March 2020. Both countries, according to the WOAH report, regained ASF-free status [25]. However, this no longer applies to the Czech Republic, which, in December 2022, confirmed ASF again on its territory and, to date, has not been able to control the disease [27].

ASF was first confirmed in Poland in February 2014, in the eastern part of the Podlaskie voivodeship, near the border with the Republic of Belarus, where the carcass of a dead wild boar was found. The presence of ASFV genetic material was confirmed by the National Reference Laboratory for ASF at PIWet-PIB in Puławy [28,29,30].

**Figure 1:**
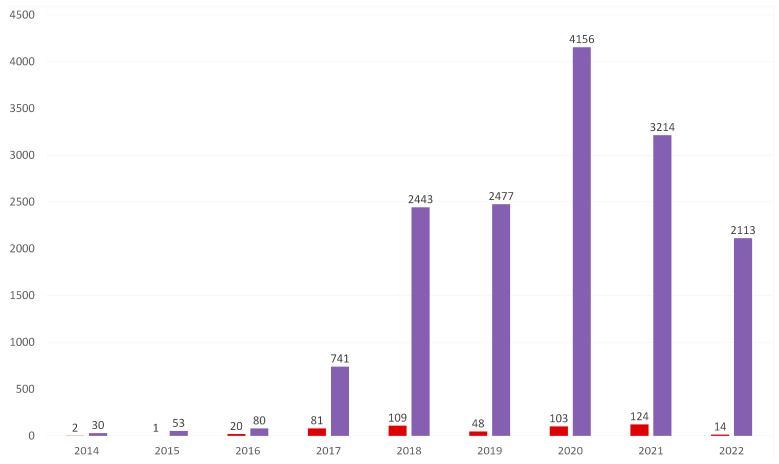
The occurrences of ASF in Poland between 2014 and 2022; red—outbreaks in pigs, purple—outbreaks in wild boars [38].

By 2016, the disease was spreading slowly, but consistently, across our country, covering two other eastern voivoideships—Podlaskie and Lubelskie—and the centrally located Mazovia, in turn. The appearance of ASF in the territory of Mazovia, at a distance of at least 100 km from the nearest outbreaks of ASF in wild boars, was most likely due to human activity [31]. In 2018, the disease crossed the northern border of the Warmian–Masurian and Subcarpathian provinces [32,33]. It is estimated that, in 2019, 5 years after the detection of the first ASF outbreak in wild boars, nearly 25% of the country’s area was occupied by disease zones [34,35]. The number of outbreaks gradually increased and reached maxima for wild boars and pigs in 2020 and 2021, respectively, but later, some declines were observed [36,37] (Figure 1).

In 2019, following the detection of the first cases of ASF in wild boars in the Lubuskie voivodeship (located approximately 300 km away from the nearest ASF case), passive surveillance (searching for wild boars dead due to ASF) and active surveillance intensified, including shooting carried out in three voivodeships: Lubuskie, Wielkopolska, and Lower Silesia. As a result, at the end of February 2020, 878 ASF outbreaks were confirmed among ‘found dead’, and ‘shot’ wild boars. From the onset of the disease in Poland in 2014 until the end of 2022, a total of 502 outbreaks were confirmed in pig herds, and 15,307 cases were found in wild boars [38]. Despite the implementation of significant restrictions and biosecurity measures in pig farming, the disease continues to spread and poses a real risk to domestic pork production. Due to human activity, it is also important to remember the possibility of the virus being carried over long distances, into regions/countries previously free of ASF.

## 3. Etiological Agent

ASFV is a large, enveloped DNA virus, whose replication cycle takes place in the cell nucleus and cytoplasm (nucleocytoplasmic large DNA viruses, NCLDV). It is classified as a member of the family *Asfarviridae*, genus *Asfivirus*. Its multilayered virion shape is icosahedral and reaches about 260 to 300 nm in size [39,40]. At the centre of the virion is a nucleoid containing genetic material, in the form of a double-stranded DNA (dsDNA) enclosed by a nuclear protein core, an internal lipid envelope, a capsid, and an external lipid envelope. The capsid forming the most external layer of the virion is made up of 2760 hexameric and 12 pentameric protein capsomeres. The previously mentioned external lipid envelope is formed upon leaving infected host cells of monocyte–macrophage lineage [39,41,42].

The ASFV genome is a 170–193 kbp long dsDNA that contains a total of 151 to 167 open reading frames (ORFs) on both strands. The central part of the genome, approximately 125 kbp long, is the central conserved region (CCR), which contains a 400 bp long variable fragment, called the central variable region (CVR). The CCR is bound by two variable regions: the left variable region (LVR) and the right variable region (RVR). They are characterised by high genetic variability, while determining the final length of the genome [42,43].

## 4. Host Range and ASFV Vectors

Species susceptible to the ASFV infection belong to the swine family (*Suidae*), including domestic pigs (*Sus scrofa f. domestica*), wild boars (*Sus scrofa*), warthogs (*Phacochoerus aethiopicus*), and red river hog (*Potamochoerus porcus*) and wild scrub pigs (*Hylochoerus meinertzhageni*) [4]. Additionally, ASFV infection was confirmed in endemic wild species in the Asia–Pacific region, including in the bearded pig (*Sus barbatus*) and Philippine warty pig (*Sus philippensis*) [44]. 

The introduction of ASFV into domestic pig or wild boar populations is characterised by high infectivity and mortality, but the disease can also transition from an epidemic to an endemic form, as is currently the case in Latvia and Estonia, and as has occurred in Sardinia since the 1970s. Although Sardinia reported its last ASF outbreak in pigs in 2018 and in wild boars in 2019, its status as an ASF-free region has not been officially confirmed. [45,46,47,48,49]. In other animals (warthogs, river pigs, and wild scrub pigs), the disease can be subclinical and, consequently, these species serve as reservoirs of the virus in the environment [50]. The presence of the ASFV, and the possibility of its replication, in soft ticks of the *Ornithodoros* spp. genus that are common in Africa and the southern part of Europe (Portugal, Spain, Italy, and Greece) has also been proven [51]. In the so-called forest cycle (otherwise known as the sylvatic cycle), proven to take place in Africa, ticks become hosts for the virus as a result of ingesting infected blood from warthogs. They can also be a biological vector, transmitting it within arthropods themselves via trans-stadial, transovarian, or sexual routes. In the aforementioned arthropods, ASFV remains infectious from several months up to 8 years [52], so soft ticks are natural reservoirs of the virus in the environment.

In Europe, the main reservoir of the virus is the Eurasian wild boar. In the wild boar population, the virus spreads in two ways: through direct contact between infected animals or through contact with the carcasses of dead wild boars or other sources of meat from infected animals, e.g., rubbish bins [53,54]. The virus can also spread over short distances through aerosol routes [55]. Due to the seasonality of infections in breeding pigs, the role of insects in the spread of the disease has been recently investigated [56,57]. To date, the transmission of the virus via hard ticks (*Ixodes ricinus* and *Dermacentor reticulatus*) found in Poland and Central European countries has not been proven, although the genetic material of the virus has been detected in them up to 8 weeks after blood collection [58]. Similarly, in the case of the common fly and mosquitoes in the European climate, e.g., the stable fly (*Stomoxys calcitrans*), the house fly (*Musca domestica*), flies of the genus *Drosophila spp.*, and mosquitoes (*Culicidae*), despite the presence of vestigial amounts of ASFV genetic material on the surface of the insects, infection of pigs can occur only by eating insects that have collected blood containing ASFV within 12 h [57]. Therefore, it should be concluded that, in temperate climates, flies and mosquitoes are not important vectors in the spread of ASFV. A significant role in the spread of the disease may be played by the human factor, as evidenced in the cases of ASF outbreaks in wild boars in areas previously free of the disease, several hundred kilometres from the nearest confirmed outbreaks of ASF in wild boars or pigs (Mazovia voivodeship and Lubuskie voivodeship). It is, therefore, reasonable to assume that the cause of the spread of ASF over such distances was, e.g., the contaminated wheels of lorries transporting animals or contaminated meat left in the forest, upon which wild boars had fed. Based on data related to these animals’ behaviour and movement, it is known that their migration to areas 20 km or more away is not realistic [59,60].

## 5. Susceptibility of ASFV to Physical and Chemical Agents

The susceptibility and stability of ASFV have been the subjects of research by many scientists over the years [61,62,63,64,65,66,67]. ASFV has been shown to possess high resistance to environmental conditions and remain infectious for a long time at temperatures below 0 °C. Similarly, under deep freeze (−70 °C) conditions, the virus is able to survive for many years without significant losses of titre and infectivity. A systematic reduction in ASFV titre was observed when ASFV-contaminated meat was frozen at −20 °C; however, the virus retained infectivity under these conditions for at least 2 years [65]. Furthermore, ASFV can survive multiple freeze–thaw cycles and remains stable in the pH range of 4 to 13. It can also remain infectious for more than an hour at 56 °C [65]. The process of curing or drying ASFV-contaminated meat (a process similar to that undergone by Parma, Iberian, or Serrano ham) allows the virus to survive in the ham for more than a year [64]. Due to its high stability, the virus is able to survive for long periods of time in meat or food swill, which thus play carrier roles in the long-distance spread of the disease. This is one of the most common routes of ASFV introduction into disease-free territories. For example, the ASF outbreak in Georgia in 2007 was caused by feeding pigs with virus-contaminated food swill from a ship that arrived from Africa [68,69]. The persistence of ASFV in the carcasses of dead, infected wild boars is a subject of discussion [70]; however, due to the high virus load in the carcasses, its role in virus transmission cannot be neglected. Consequently, controlling the spread of the disease is extremely difficult due to, among other factors, the need to actively search for dead wild boar carcasses [71]. Furthermore, the time of year has been shown to have a considerable effect on the rate of decomposition of the carcasses of fallen wild boars [72,73]. With an increase in the ambient temperature, the period of debris decomposition becomes shorter, ranging from 8 days in summer to 37 days in winter. This may affect the seasonality of ASF occurrence in wild boars, among which the highest number of outbreaks is recorded during the winter season [72,73,74,75]. The high survival rate of ASFV is also an important factor in farm pigs. It has been proven that the excreted virus can remain infectious, in the case of faeces, for 8 days at 4 °C and 3–4 days at 37 °C, and in the case of urine, for up to 15 days at 4 °C, 5 days at 21 °C, and 2–3 days at 37 °C [62].

In addition, a study by Olesen et al. [71] confirmed the possibility that pigs could become infected when entering a pen, a day after the removal of sick animals. In such a scenario, the exposure of pigs to an environment contaminated with ASFV-containing excreta can be the cause of indirect infection. For example, in Spain, ASFV was found in piggeries where sick pigs had been killed 4 months earlier [76]. Therefore, excreta containing ASFV should be considered an important factor in the spread of the virus, especially within the herd and farm [67].

ASFV can be effectively inactivated with high temperature (60 °C/20 min. or 56 °C/70 min.), changes in pH (<3.9 or >11.5), or the use of disinfectants with proven efficacy [77].

## 6. Disease Control: Combating ASF through Administrative Methods

**Figure 2:**
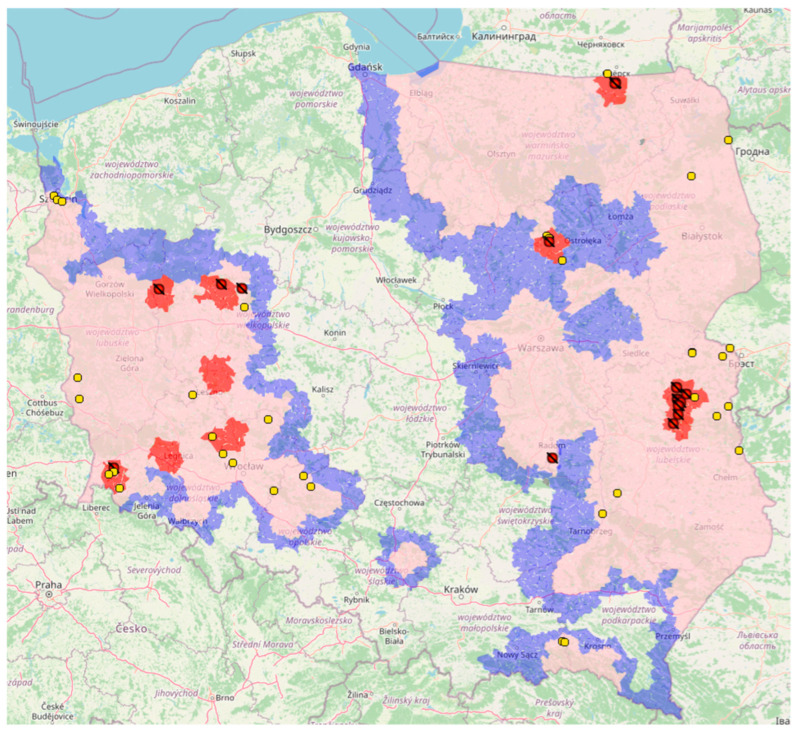
The distribution of restricted zones and ASF outbreaks in pigs (red dots) and wild boars (yellow dots) in Poland in 2023 (state as of July, 2023) [82].

ASF is considered one of the most dangerous swine diseases, negatively affecting the pig-producing sector, and, therefore, it was included in the WOAH list of notifiable diseases; its control is strictly regulated in the EU and multiple countries outside of Europe [78]. The measures applied include surveillance, epidemiological investigation, and the control of animal movements (in both pigs and wild boars), as well as wild boar hunting and the elimination of pigs affected by ASF outbreaks. Strict adherence to the principles of quarantine and biosecurity, as well as the use of disinfectants with proven efficacy, is indispensable [79]. There is no universal scenario for the approach to disinfection of the area (environment) where the outbreak was detected, due to the different places where ASF carcasses may be found (forests or farm fields in the case of wild boars, or farms in the case of domestic pigs). If the disease is confirmed on a farm, all pigs are slaughtered and disposed of, while the facilities where the animals were housed are subject to cleaning and disinfection with biocides that are virucidal against ASFV [80]. Veterinary inspection takes steps to determine the source of the infection and the possibility of spreading the disease to more farms or other entities (e.g., meat rendering plants and feed companies). It also verifies that biosecurity measures were followed in each case, as this is required to receive compensation payments. According to the guidelines of the European Commission, some regulations related to the marking of ASF zones have been introduced [81]. Regulations related to the control of disease outbreaks have not changed. Currently, restricted area I is marked in blue, restricted area II is marked in pink, and restricted area III is marked in red. A red zone (with a minimum radius of 3 km) and a pink zone (extending at least 7 km beyond the red zone) are designated around the ASF outbreak (Figure 2) [35,82]. Farms located in these areas are banned from moving pigs for 40 days and 30 days, respectively, and after this time, the movement of animals from the farms can take place only after obtaining a permit from the District Veterinarian. In addition, in restricted areas, a laboratory examination of the herd must be carried out within 15 days before the planned movement, as well as a clinical examination 24 h before the animals are slaughtered (blue and red zones); in the case of the pink zones, tests carried out before the animals themselves are moved and slaughtered are sufficient. Resettlement of a farm where an outbreak of ASF in pigs was previously confirmed is possible 40 days after the completion of disinfection procedures. However, after resettlement, the new pig herd is subjected to serological testing for the detection of antibodies to ASFV [83,84]. 

## 7. Prevention—Monitoring and Regulation of Wild Boar Populations

Administrative actions must be supported by a program to monitor the epizootic situation in both the pig and wild boar populations. Experience in detecting ASF indicates that passive surveillance, i.e., searching for and examining wild boar carcasses, is the most effective in the latter regard. The percentage of carcasses positive for the presence of ASFV genetic material in the red zone area (restricted area III) was more than 60%, while active surveillance (examination of shot wild boars) showed about 0.8% positive results in the same area, proving that this method was much less useful for determining the current epidemiological status related to ASF in the wild boar population [85]. However, active surveillance is an important part of a comprehensive ASF surveillance plan, as the detection of the virus in wild boars that do not show clinical signs yet allows for the early detection of the disease in new areas, followed by rapid intervention, and consequently reduces the risk of virus transmission. It is also important to conduct serological monitoring, among wild boars that have been shot, to determine the character of the disease’s course in the population (e.g., transition from epizootic to endemic form). In countries where ASF has been present for more than 8 years (Estonia and Latvia), the percentage of seropositive wild boars is more than 20%, which differs from the situation in Poland, where it remains at a level of 1.5–2% [74].

A growing population of wild boars, which represent the main reservoir of ASFV in the environment, increases the chances of the virus spreading into new geographical areas and the long-term persistence of the disease in the wild, thus escalating the risk of its introduction into the domesticated swine population. The example of the Czech Republic, where the directed reduction in the wild boar population around affected areas allowed them to quickly regain ASF-free status (since 2018), proves that the intensive hunting of wild boars is capable of rapid eradication of the disease. Unfortunately, in December 2022, ASF returned to the Czech Republic, where it was again reported in the wild boar population [80]. These measures are, thus, only effective in the case of a “spot” introduction of the pathogen into the wild boar population. The combination of control measures implemented in Belgium (an ASF-free country since 2020), including fencing, sniper-guided shooting and trapping of wild boars, and, most importantly, searching for and disposing of wild boar carcasses, with their intensities adapted to the epidemiological situation, is currently considered effective for ASF control [86]. Despite the controversy surrounding the radical depopulation of wild boars, this is one of the most effective and recommended measures, among others, by the European Food Safety Authority (EFSA), to eradicate the disease from wildlife animal populations. However, taking into account the experiences of ASF-affected countries, the application of a fully effective wild boar depopulation strategy is not possible.

## 8. Perspectives on Developing a Vaccine against ASF

Even though the work on a vaccine against ASF has been ongoing for decades, a safe, commercially available vaccine has not yet been developed. The problems with developing a vaccine are related to the complex structure of the ASFV, its ability to evade the host immune response, and the lack of induction of antibodies that fully neutralise the virus in infected or vaccinated animals. Another challenge is developing a strategy through which to reliably differentiate vaccinated from infected animals (DIVA strategy, or differentiating infected from vaccinated animals), based on compatible diagnostic tests (molecular and serological) [87,88]. Intensive efforts and repeated attempts to use, e.g., homogenates from cultures of cells infected with ASFV, supernatants containing infected peripheral blood leukocytes, purified inactivated ASFV virions, inactivated strains with and/or without adjuvants, attenuated strains, and vaccines based on peptide constructs, failed to achieve sufficient post-vaccination protection against ASF in experimentally vaccinated and infected pigs [89,90,91,92,93,94,95,96]. The most promising results were obtained with genetically modified attenuated strains, as they induce a cell-mediated immune response. 

In 2020, there was a breakthrough in the research on an ASF vaccine. A team of scientists from Plum Island (United States of America, USA), published the results of a study on a vaccine (ASFV-G-ΔI177L) based on a virus strain with deletion of the 177L gene. Currently, this ASFV deletion strain is the most promising candidate for the development of a vaccine against ASF, as it induces immunity against both Asian and European variants of the ASFV. During the study, it was observed that one-third of the tested pigs showed immunity against ASFV the second week after vaccination, while complete immunity of all pigs was achieved four weeks after the application of the experimental vaccine [87]. In 2021, the same team also conducted a study on the administration of an experimental vaccine via the oral–nasal route. The results obtained proved that pigs vaccinated via this route showed immunity to ASFV infection, giving hope for the development of an effective vaccine for wild boars in the future [97]. There are at least two more serious vaccine candidates from Germany and China. The first candidate, “ASFV-G-∆MGF”, was used for both oral delivery in wild boars and intramuscular delivery in domestic pigs. However, while, in pigs, the vaccine candidate induced full immunity after two inoculations, in wild boars, 50% seroconverted, and within the remaining group of animals, two developed acute lethal infection and two a mild and transient course of the disease [98]. Another prototype vaccine from China—HLJ/18-7GD—obtained through the deletion of seven genes, provided complete immunity against lethal ASF infection after the challenge. Additionally, no signs of possible reversion to virulence were observed in pigs, but this prototype still needs to be tested on wild boars, which represent a major problem in the spread of ASF in European areas [99]. Despite promising results, the developed vaccine prototypes still need to pass a number of tests for safety, side effects, potential stability, and, most importantly, efficacy in field conditions before they can be released commercially. Furthermore, to bring the vaccine to the market, current EU legislation prohibiting treatment and vaccination against ASF must be changed. However, recent news reports that the Vietnamese government has commercialised two domestic vaccines against ASF: NAVET-ASFVAC, based on ASFV-G-ΔI177L, and AVAC ASF LIVE, based on the ASFV-G-Δ-MGF strain [100]. Unfortunately, recent reports on the three new genotype I and II ASF recombinants detected in China do not suggest any reason for optimism, as they may pose additional significant problems for a vaccine’s efficacy [101]. Therefore, at present, early recognition of the disease, reliable and rapid laboratory diagnosis, administrative depopulation of pig herds in which ASF has been found, and the application of biosecurity measures defined by an official eradication approach play key roles in the control of ASF [102,103].

## 9. Biosecurity of Swine Herds 

In the absence of an effective and commercially available vaccine, biosecurity is extremely important, and, in fact, represents the only effective measure in preventing the spread of ASFV infection [104]. Adherence to its principles strengthens the protection of farms against infection, in addition to limiting the spread of the disease in the environment. Years of observation, as well as previously developed biosecurity models, have shown that the application of even basic biosecurity standards has noticeably reduced and limited the spread of ASFV [11,105,106]. Biosecurity is not only broadly defined as biosafety with respect to buildings, rules of movement within the facility, and agricultural equipment, but also, more importantly, the awareness of people and the public about the scale of the threat and possible consequences associated with the occurrence of infectious disease. Precise, accessible guidelines, training, and support are the basis for the correct application of the principles and rules of biosecurity. In terms of farm protection, there are two types of biosecurity, i.e., external and internal. External biosecurity refers to the measures aimed at securing the farm against the introduction of pathogens from the outside environment, and they include, among other things, fencing the farm, keeping appropriate distance between farms, required quarantine before the targeted introduction of new animals into the herd, minimising the visits of unauthorised persons to the farm, washing and disinfecting every vehicle that enters, protecting feed from animal access, and protecting windows from access by wild animals, rodents, fowl, or even insects, which can sporadically become mechanical vectors for the disease. Internal biosecurity includes management aimed at limiting the possibility of spreading the disease within the farm area, and it involves disinfecting facilities, regulating the flow of animals according to the principle of “all in/all out”, separating dirty and clean zones, changing protective clothing, using separate equipment for clean and dirty zones, and prophylactic vaccination and management of the herd through the implementation of preventive programmes [107,108,109,110,111]. One of the key principles of effective biosecurity is the use of effective disinfection techniques and the adherence to established disinfection protocols by farm personnel and farm visitors, including veterinarians [112,113].

## 10. Disinfection

Disinfection, supported with a mechanical cleaning process, is fundamental in deactivating swine pathogens, prevents the spread of infectious diseases, and is required before the repopulation of a farm after an outbreak [114]. The complete disinfection process should include two steps: thorough mechanical washing and proper disinfection. Potentially contaminated materials, such as manure, bedding, straw, and feed, should be removed and disposed of. The surfaces of floors and walls should be thoroughly washed with detergents, and then dried and disinfected [114,115]. The optimal disinfectant should be characterised by fast action, stability, a lack of toxicity, and environmental resistance, and, more importantly, it should have the broadest possible biocidal spectrum, fighting bacteria, viruses, and fungi. Incorrectly selected parameters of the agent used in disinfection (e.g., concentration, contact time, and/or range of application) can lead to ineffective operation and the failure of disinfection processes. Only authorised disinfection products, with proven efficacy against ASFV, should be used during this process, according to the manufacturer’s instructions [111,112,116].

Due to the lack of precisely described and detailed data on disinfectants against ASFV, it is difficult to recommend an ideal biocidal agent. However, some EU member states have developed a list of authorised biocides that are effective against ASFV. This classification was based on general knowledge and experience in the use of disinfectants against enveloped viruses, such as the equine arteritis virus (EVAV), Aujeszky’s disease virus (PRV), the porcine reproductive and respiratory syndrome virus (PRRSV), and the classical swine fever virus (CSFV). So far, chemical compounds considered effective in inactivating ASFV include the following:

− 1% formaldehyde;
− sodium hypochlorite (0.0075% to 0.03%);
− 2% caustic soda solution (the most potent virucide);
− glutaraldehyde, formaldehyde;
− 1% sodium or calcium hydroxide (inactivation of the virus in suspension at 4 °C);
− phenols, such as Lysol and creolin;
− lipid solvent-based chemicals;
− multicomponent compounds—such as Virkon (1:100), Lysoformin, Desoform, and OD 20—surfactants, active substances, organic acids, glycosal [116,117,118,119], and others.

The compounds mentioned above, although never previously tested, are recommended as effective against this virus and are commonly used in the production of commercially available disinfectants. The assumption of an agent’s virucidal efficacy against ASFV based on experiences with other viruses may be burdened with error and lead to disastrous consequences related to an ineffectiveness of the crucial biosecurity process of disinfection and, consequently, an increased risk of ASF spread. Despite their great importance in controlling the spread of the disease, until recently, little information was available on the effectiveness of chemical compounds against ASFV. Related research results published over the years mainly concern the efficacy of selected active substances against ASFV on different types of surfaces [66,120,121,122,123]. To clarify doubts about the effectiveness of disinfectants used so far and to confirm their virucidal activity against ASFV, several studies have been carried out [79,113,124,125,126]. The highest virucidal efficacy against ASFV was shown for sodium hypochlorite, which was effective even in a concentration of 0.3% and under high-level soiling conditions [79,113]. Chlorine has been proven to play a key role in its composition, and it must oscillate in the range of 0.5%. Long-term storage lowers the level of active chlorine, reducing the disinfection effectiveness of sodium hypochlorite [116,127]. An equally strong chemical compound that inactivates ASFV is caustic soda, which, in a study by Juszkiewicz et al. (2020), was reported to cause the inactivation of ASFV at final concentrations of 1%, 2%, and 3%, with the exception of the lowest concentration under high soiling conditions [79]. However, in the case of calcium hydroxide, its ability to inactivate ASFV is related to a different temperature range during disinfection. The best virucidal efficacy against ASFV was obtained at concentrations of 0.2%, 0.5%, and 1%, at a temperature of 22 °C, while, at a temperature of 4 °C, it was effective only at concentrations of 0.5% and 1% [66]. Potassium peroxymonosulfate, phenol, and benzalkonium chloride were tested at the same concentrations (0.5%, 1%, and 2%). All three chemical compounds were effective at a concentration of 1%, irrespective of soiling level, with the exception of benzalkonium chloride, which showed virucidal activity at the highest concentration tested (1%) only at a low soiling level. Glutaraldehyde was highly virucidal against ASFV at all concentrations tested at both levels of soiling. As the research showed, ASFV was effectively inactivated with commercially available disinfectants based on iodine, sodium hypochlorite, and potassium peroxymonosulfate [113,124]. A significant innovation in the topic of disinfection was the study of plant extracts and their virucidal capacity against ASFV, during which the effectiveness of peppermint against this virus was proven [128]. The virucidal activity of natural plant extracts may, in the future, facilitate the development of more ecologically friendly disinfectants.

The WOAH recommends a series of disinfectants for the inactivation of ASFV: 8/1000 sodium hydroxide (30 min), 2.3% hypochlorite (3 min), 3/1000 formalin (30 min), 3% n-phenyl phenol, and an iodine compound (30 min) [129]. Despite the recommendations of WOAH, the study by Juszkiewicz et al. (2020) failed to assess the effectiveness of formaldehyde against this virus, due to its high cytotoxicity for cell cultures [79,127]. 

In addition to chemical disinfection, ASFV can be inactivated at 60 °C for 30 min and at pH levels <3.9 or >11.5 in a serum-free medium [130,131]. Ozonised water also exhibits a virucidal effect against ASFV, according to a study by Zhang et al. (2020). However, only 5 mg/L of ozonised water was able to reduce the virus titre to the required disinfection standards of 4 log_10_ [132].

## 11. Summary

As a result of continuous ASF expansion, totals of 523 cases in domestic pigs and 17,391,502 in wild boars were confirmed in Poland by the end of July 2023, resulting in economic losses that are difficult to estimate [82]. The lack of a commercially available vaccine against ASF limits the ability to control the spread of the disease through administrative measures by stamping out and disposing of pigs in which disease is found [93]. Currently, the only measures of preventing ASF include the implementation and adherence to strict biosecurity rules. One of the key elements of properly implemented biosecurity is effective disinfection [117]. Although some countries have a list of virucidal products approved for use against ASFV, their effectiveness was mostly estimated indirectly, based only on testing them against other enveloped viruses. The lack of data available so far on the evaluation of the efficacy of disinfectants against ASFV, resulting in the common use of agents with unconfirmed efficacies against this virus, may lead to the uncontrolled spread of the disease. However, in recent years, some studies have attempted to solve this problem. According to the available research, the virucidal efficacy against ASFV has been confirmed for eight active substances, including formaldehyde, sodium hypochlorite, caustic soda, glutaraldehyde, phenol, benzalkonium chloride, potassium peroxymonosulfate, and acetic acid [79]. Most of these chemicals inactivated the virus at the concentrations recommended by the WOAH [127]. The highest effectiveness levels were demonstrated with sodium hypochlorite, glutaraldehyde, caustic soda, and potassium peroxymonosulfate. The least effective disinfectant against ASFV was benzalkonium chloride. In addition, the great importance of pre-cleaning steps, preceding an actual disinfection, in order to remove contaminants, has been confirmed [79]. Due to the serious health consequences that can occur during long-term exposure to the toxic effects of chemical disinfectants, there was an additional effort to investigate the effectiveness of plant extracts in order to identify a safe and effective plant-based alternative. Fourteen plant extracts were selected for the study. The results showed that only peppermint extract exhibited high virucidal activity against ASFV [128]. 

The abovementioned research has, for the first time, allowed for the identification of the most effective chemical substances and conditions ensuring the effectiveness of disinfection processes against ASFV. An additional element of this innovation was the determination of virucidal activity in natural plant extracts, which may, in the future, be among the components of ecological disinfectants.

Until a safe vaccine against ASFV is approved, biosecurity and effective disinfection are the most important measures through which to prevent the spread of ASF. Training pig farmers to reliably adhere to biosecurity rules, and educating them about the effective use of disinfectants, is of paramount importance. Providing farmers with this knowledge reinforces the control of diseases like African swine fever, safeguarding the health and welfare of the animals involved. Implementing appropriate biosecurity practices and using effective disinfectants significantly reduce the risk of disease transmission, ensuring the stability and sustainability of the swine farming industry.

## Data Availability

Not applicable.
